# The relationship between proton pump inhibitor use and serum magnesium concentration among hemodialysis patients: a cross-sectional study

**DOI:** 10.1186/s12882-015-0139-9

**Published:** 2015-08-13

**Authors:** Paraish S. Misra, Ahsan Alam, Mark L. Lipman, Sharon J. Nessim

**Affiliations:** Department of Medicine, Jewish General Hospital, McGill University, Montreal, Canada; Department of Medicine, Division of Nephrology, Royal Victoria Hospital, Montreal, Canada; Department of Medicine, Division of Nephrology, Jewish General Hospital, 3755 Cote-Sainte-Catherine Road, Room G-225.1, Montreal, QC H3T 1E2 Canada

## Abstract

**Background:**

Observational data suggest that serum magnesium (Mg) concentration is inversely related to vascular calcification and hyperparathyroidism among patients with end-stage renal disease (ESRD). In recent years, there have been several case reports of hypomagnesemia due to use of proton-pump inhibitors (PPI), with the hypomagnesemia attributed to inappropriate gastrointestinal (GI) Mg loss. We hypothesized that the tendency to GI Mg loss is more common than is currently reported. Since patients with ESRD have little to no renal Mg loss to affect serum Mg concentration, dialysis patients are an interesting population in whom to study the relationship between PPI use and serum Mg levels.

**Methods:**

Using a single-center cross-sectional design, we studied 155 prevalent hemodialysis (HD) patients. Serum Mg concentration for each patient was determined based on the mean of 3 consecutive serum Mg levels drawn at 6 week intervals. PPI use at the time of the blood tests was documented. The relationship between PPI use and Mg concentration was determined in unadjusted analyses, as well as after adjustment for age, gender, race, cause of ESRD, diabetes, time on HD and dialysate Mg concentration.

**Results:**

55 % of patients were on PPIs at the time of the study. The majority of patients (62 %) used a dialysate Mg (in mmol/L) of 0.5, and the remainder (38 %) used a dialysate Mg of 0.375. Serum Mg levels were significantly lower among PPI users vs. non-users (0.93 vs. 1.02 mmol/L, p < 0.001). This finding persisted after stratifying for dialysate Mg concentration, and after multivariable adjustment (p < 0.001). In addition, more PPI users vs. non-users had a Mg level < 1 mmol/L (79 % vs. 43 %) and a Mg level < 0.8 mmol/L (16 % vs. 4 %). There was a non-significant trend toward increased time on PPI being associated with lower serum Mg levels (p = 0.067).

**Conclusion:**

Among HD patients, PPI users have lower serum Mg levels as compared with non-users. Further research is required to determine whether the magnitude of change in Mg levels among PPI users is associated with adverse outcomes.

## Background

In recent years, there have been several case reports of hypomagnesemia due to proton-pump inhibitor (PPI) use [1–6]. In each of these cases, the hypomagnesemia was identified because it was severe enough to cause symptoms. As a result of these reports, in 2011, the Food and Drug Administration in the Unites States sent out a Drug Safety Communication warning that low magnesium (Mg) levels may be associated with long-term use of PPIs. In these case reports of PPI-induced hypomagnesemia, renal excretion of Mg was appropriately low, suggesting that the etiology is related to GI loss of Mg through a mechanism that has not yet been fully elucidated. While this may be an idiosyncratic reaction, it is plausible that the high gastric pH present in PPI users may alter Mg transport resulting in a tendency to GI Mg loss in all patients using PPIs. If this is the case, the development and severity of hypomagnesemia would depend on a combination of the extent of GI Mg loss and Mg intake.

Some observational data have started to emerge linking PPIs with hypomagnesemia [7–10], although the data are conflicting [11, 12]. Several factors make the hemodialysis (HD) population well suited to study the relationship between PPI use and serum Mg concentration. First, HD patients have little or no urine output, which reduces potential confounding related to renal Mg loss. Furthermore, HD patients are typically dialyzed against an ionized Mg concentration of 0.5 mmol/L or less in the dialysate (corresponding to a total Mg concentration of approximately 0.7 mmol/L or less), such that the dialysate Mg concentration would be insufficient to raise serum Mg in most instances.

We therefore hypothesized that mean serum Mg levels among HD patients using PPIs would be lower than levels among HD patients not on PPIs, and that increased ‘time on PPI’ would be associated with lower serum Mg levels.

## Methods

### Patient population and study measurements

This was a cross-sectional study of 155 prevalent HD patients at the Jewish General Hospital, a tertiary care academic hospital in Montreal, Quebec, Canada. All HD patients in our program in February of 2011 were considered for inclusion in the study. Standard HD therapy consisted of 4-hour treatments 3 times weekly using a high flux dialyzer. Patients were excluded from the study if they were receiving dialysis for acute kidney injury (within 3 months), if they were hospitalized at any time over the 3-month period during which the blood tests were drawn, or if they had a history of chronic diarrhea, or an ileostomy or colostomy. None of the patients were receiving Mg-based phosphate binders or other Mg-based medications. Serum Mg concentration for each patient was determined based on the mean of 3 consecutive serum Mg levels drawn at 6 week intervals as part of routine dialysis blood testing. Data on PPI use was extracted from the dialysis pharmacy database, and time on PPI at the time of Mg measurement was also recorded.

The relationship between PPI use and serum Mg was determined in unadjusted analyses, as well as after adjustment for age, gender, race, cause of ESRD, diabetes, time on HD and dialysate Mg concentration. The primary endpoint was the difference in the mean Mg concentration in the presence or absence of PPI use. Secondary endpoints included the proportion of HD patients with Mg levels below various cutoffs in the presence or absence of PPI use, and the relationship between ‘time on PPI’ and serum Mg concentration. Approval was obtained from the Research Ethics Board at the Jewish General Hospital prior to study initiation. No written informed consent was obtained based on the study’s retrospective nature.

### Statistical analysis

Continuous data are presented as mean +/− standard deviation, or median and interquartile range as appropriate. Continuous variables were compared using the Student’s *t*-test for normally distributed data or the Mann–Whitney test for non-normally distributed data, and using the chi-square test for categorical variables. A multivariate linear regression model was used to determine the independent effect of PPI use on Mg levels after adjusting for age, gender, race, diabetic status, cause of ESRD, time on HD and dialysate Mg. Among PPI users, a multivariate linear regression model was used to determine whether time on PPI was predictive of serum Mg level. Statistical significance was defined as a p value of <0.05. All statistical analyses were performed using SAS (version 9.3).

## Results

A total of 180 patients were screened for inclusion, with 25 excluded due to either initiation of dialysis for acute kidney injury, hospitalization during the time of data collection or presence of chronic increased GI losses. Among 155 HD patients included in the study, mean age was 70 years, with a female and Caucasian predominance (Table 1). Fifty five percent of patients were on PPIs at the time of the Mg measurements, and median time on the PPI was 15 months (interquartile range 4–25 months). The majority of patients (62 %) used a dialysate Mg of 0.5 mmol/L, and the remainder (38 %) used a dialysate Mg of 0.375 mmol/L.

**Table 1:** Patient Demographics

	PPI users (n = 86)	PPI non-users (n = 69)	P value
Age (mean, years)	72 ± 12	67 ± 17	0.05
Gender (% male)	33 %	30 %	0.78
Race (%)			0.09
Caucasian	74 %	64 %	
Black	16 %	15 %	
Asian	9 %	17 %	
Other	0 %	4 %	
Cause of ESRD			0.12
Hypertension	27 %	25 %	
Diabetes	50 %	36 %	
Glomerulonephritis	10 %	16 %	
Polycystic kidney disease	0 %	4 %	
Other	13 %	18 %	
Diabetic (%)	52 %	39 %	0.10
Dialysate Mg concentration (%):			0.28
0.5 mmol/L	58 %	67 %	
0.375 mmol/L	42 %	33 %	
Time on HD (in months [IQR])	36 (20–63)	26 (16–43)	0.02

**Fig. 1:**
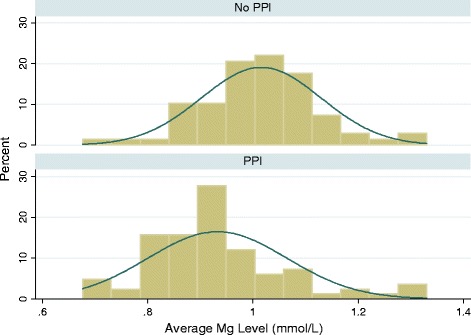
Histogram of serum magnesium levels among PPI users vs. non-users

Serum Mg levels were significantly lower among PPI users vs. non-users (0.93 vs. 1.02 mmol/L, p < 0.0001) (Fig. 1). This finding persisted even after stratifying for dialysate Mg concentration. More PPI users vs. non-users had serum Mg levels < 1 mmol/L (79 % vs. 43 %, p = 0.0003), and serum Mg levels < 0.8 mmol/L (16 % vs. 4 %, p = 0.02) (Table 2). On multivariate analysis, after adjustment for age, gender, race, cause of ESRD, diabetes, time on HD and dialysate Mg concentration, PPI use remained an independent predictor of lower serum Mg (p = 0.0001). Among PPI users, there was a trend toward increased time on PPI being associated with lower serum Mg levels, but this did not reach statistical significance (p = 0.067).

**Table 2:** Percentage of patients with low Mg levels

	PPI users (n = 86)	PPI non-users (n = 69)	P value
Patients with Mg ≤1.0 mmol/L n (%)	68 (79)	30 (43)	<0.001 (exact p value is 0.0003)
Patients with Mg ≤0.8 mmol/L n (%)	14 (16)	3 (4)	0.018

## Discussion

In this cross-sectional study of prevalent HD patients, we found that serum Mg levels among PPI users were significantly lower than Mg levels of those who were not on PPIs, even after adjustment for relevant covariates. These results suggest that the effect of PPI use on GI loss of Mg is likely present in a substantial proportion of patients, and may therefore be an under-recognized entity that only comes to clinical attention when the hypomagnesemia is severe enough to cause symptoms.

Since the emergence of numerous case reports of PPI-induced hypomagnesemia over the last decade, increasing attention has been paid to this issue. Several observational studies have been published recently, with a majority reporting on the relationship between PPIs and Mg levels in hospitalized patients [7–9, 11, 13], and a few others studying this association in various outpatient settings [10, 12, 14]. Among the studies in hospitalized patients, all but one [11] showed a significant relationship between PPI use and lower serum Mg levels, and two of the five studies in hospitalized patients showed that hypomagnesemia was most pronounced in patients concurrently on PPIs and diuretics [7, 9]. The observational studies that focused on non-hospitalized patients included one in a large cohort of stable outpatients that showed that PPI use in the 4 months prior to Mg measurement was independently associated with hypomagnesemia [14]. In contrast, another outpatient study performed in renal transplant recipients showed no relationship between PPIs and Mg levels [12]. However, the latter study also failed to demonstrate any association between Mg levels and either diabetes or diuretics, both well established etiologies of hypomagnesemia. One of the most recent studies looked at 62 male HD outpatients from a single center and showed a significant relationship between PPI use and serum Mg levels [10]. Our study adds to this literature by validating this association in a larger and more diverse cohort of HD patients.

The basis for the Mg lowering effect of PPIs has yet to be fully elucidated, but among over forty reported cases of PPI-induced hypomagnesemia, the consistent finding of low urinary Mg levels strongly suggests that Mg depletion occurs in the GI tract. While passive absorption accounts for most of GI Mg uptake, carrier-mediated pathways involving TRPM-6 and TRPM-7 are also known to be critical to GI Mg homeostasis [15]. While it has been suggested that the major mechanism by which PPIs induce Mg wasting is through TRPM-6 and TRPM-7 inhibition [16], there is also some evidence that interference with the passive absorption mechanism may underlie this phenomenon [17, 18]. Another unexplained observation is the discrepancy between the ubiquity of PPI use and the rarity of the associated hypomagnesemia. Although this may indicate an idiosyncratic side-effect of PPI use, our results and those of others show that absolute Mg levels remain lower in PPI users, suggesting that PPIs have a consistent Mg-depleting effect in most patients. In support of this is the finding that PPI users predictably demonstrate enhanced renal Mg absorption [19], indicating chronic subclinical depletion in this population.

While the differences in Mg levels between PPI users and non-users in our study were highly statistically significant, it remains to be determined whether the lower serum Mg levels among PPI users are clinically significant. Potential adverse outcomes that have been associated with lower Mg levels in dialysis patients include hyperparathyroidism, vascular calcification and mortality. For example, Mg levels in dialysis patients have been shown in observational studies to be inversely proportional to parathyroid hormone levels in both HD [20–22] and peritoneal dialysis populations [23, 24]. In addition, Mg is known to inhibit calcification in vascular smooth muscle cells *in vitro* [25, 26], and there have been several observational studies demonstrating an association between lower serum Mg levels and vascular calcification [27–29] or mitral annular calcification [30]. Furthermore, a recent small randomized pilot study showed that supplementation with Mg carbonate for 1 year in HD patients led to improved calcification scores in some patients [31]. In this latter trial, the difference in Mg levels between the groups was small but significant (1.16 vs. 1.04 mmol/L). Finally, there are limited data suggesting an association between lower Mg levels and increased mortality among HD patients. This was first illustrated in an observational study of 515 HD patients, in whom baseline serum Mg concentration (measured over a 4 month period) predicted mortality [32]. Specifically, after a mean of 51 months of follow-up, those with serum Mg <1.14 mmol/L had a higher mortality relative to those with Mg levels ≥ 1.14 mmol/L. More recently, in a Japanese registry-based cohort study of 142,555 HD patients by Sakaguchi *et al.*, Mg concentrations in the 3 lowest sextiles (corresponding to Mg levels < 1.10 mmol/L) were associated with higher all-cause mortality, including increased cardiovascular mortality and non-cardiovascular mortality [33]. While the data to date are almost all observational, they raise the possibility that subtle differences in Mg levels even within the normal range among dialysis patients can have an impact on clinically meaningful outcomes.

While the major strength of this study is the use of a stable, outpatient population with minimal residual renal function to confound the site of Mg loss, the study has some limitations. First, it is cross-sectional in nature, and therefore does not allow for description of the evolution of Mg levels among PPI users. Second, we did not measure dietary Mg intake, markers of nutritional status, Kt/V or renal Mg loss. However, for the latter variable, given that the median time on HD was 32 months, the extent of renal Mg loss would be minimal [34] and would have been unlikely to confound the results. Third, we studied serum Mg levels, but did not specifically look at the ionized Mg fraction. Fourth, while we did not document adherence, PPI use was determined from our dialysis pharmacy record, which was updated every 2–3 months based on contact with the patients’ outpatient pharmacies. Lastly, the study may have been underpowered to detect other significant associations, such as the impact of duration of PPI use on Mg levels, which trended toward but did not reach significance in our study.

## Conclusion

In conclusion, in this single-center study using a cross-sectional design, we have shown that PPI use is associated with significantly lower levels of serum Mg in stable HD patients, suggesting that the effect of PPIs on Mg homeostasis is much more prevalent than originally thought. While we did not study the association between lower Mg levels and adverse outcomes, there is a growing literature among dialysis patients on the potential link between lower Mg levels and the risk of calcification, hyperparathyroidism and mortality. Further research is required to determine whether the magnitude of change in Mg levels among PPI users on HD is associated with adverse outcomes.

## Abbreviations

ESRD: end-stage renal disease
GI: gastrointestinal
HD: hemodialysis
Mg: magnesium
PPI: proton pump inhibitor
